# A Privacy-Preserving Distributed Medical Data Integration Security System for Accuracy Assessment of Cancer Screening: Development Study of Novel Data Integration System

**DOI:** 10.2196/38922

**Published:** 2022-12-30

**Authors:** Atsuko Miyaji, Kaname Watanabe, Yuuki Takano, Kazuhisa Nakasho, Sho Nakamura, Yuntao Wang, Hiroto Narimatsu

**Affiliations:** 1 Graduate School of Engineering Osaka University Suita Japan; 2 Japan Advanced Institute of Science and Technology Nomi Japan; 3 Cancer Prevention and Control Division Kanagawa Cancer Center Research Institute Yokohama Japan; 4 Department of Genetic Medicine Kanagawa Cancer Center Yokohama Japan; 5 Graduate School of Science and Technology for Innovation Yamaguchi University Ube Japan; 6 Graduate School of Health Innovation Kanagawa University of Human Services Kawasaki Japan

**Keywords:** data linkage, data security, secure data integration, privacy-preserving linkage, secure matching privacy-preserving linkage, private set intersection, PSI, privacy-preserving distributed data integration, PDDI, big data, medical informatics, cancer prevention, cancer epidemiology, epidemiological survey

## Abstract

**Background:**

Big data useful for epidemiological research can be obtained by integrating data corresponding to individuals between databases managed by different institutions. Privacy information must be protected while performing efficient, high-level data matching.

**Objective:**

Privacy-preserving distributed data integration (PDDI) enables data matching between multiple databases without moving privacy information; however, its actual implementation requires matching security, accuracy, and performance. Moreover, identifying the optimal data item in the absence of a unique matching key is necessary. We aimed to conduct a basic matching experiment using a model to assess the accuracy of cancer screening.

**Methods:**

To experiment with actual data, we created a data set mimicking the cancer screening and registration data in Japan and conducted a matching experiment using a PDDI system between geographically distant institutions. Errors similar to those found empirically in data sets recorded in Japanese were artificially introduced into the data set. The matching-key error rate of the data common to both data sets was set sufficiently higher than expected in the actual database: 85.0% and 59.0% for the data simulating colorectal and breast cancers, respectively. Various combinations of name, gender, date of birth, and address were used for the matching key. To evaluate the matching accuracy, the matching sensitivity and specificity were calculated based on the number of cancer-screening data points, and the effect of matching accuracy on the sensitivity and specificity of cancer screening was estimated based on the obtained values. To evaluate the performance, we measured central processing unit use, memory use, and network traffic.

**Results:**

For combinations with a specificity ≥99% and high sensitivity, the date of birth and first name were used in the data simulating colorectal cancer, and the matching sensitivity and specificity were 55.00% and 99.85%, respectively. In the data simulating breast cancer, the date of birth and family name were used, and the matching sensitivity and specificity were 88.71% and 99.98%, respectively. Assuming the sensitivity and specificity of cancer screening at 90%, the apparent values decreased to 74.90% and 89.93%, respectively. A trial calculation was performed using a combination with the same data set and 100% specificity. When the matching sensitivity was 82.26%, the apparent screening sensitivity was maintained at 90%, and the screening specificity decreased to 89.89%. For 214 data points, the execution time was 82 minutes and 26 seconds without parallelization and 11 minutes and 38 seconds with parallelization; 19.33% of the calculation time was for the data-holding institutions. Memory use was 3.4 GB for the PDDI server and 2.7 GB for the data-holding institutions.

**Conclusions:**

We demonstrated the rudimentary feasibility of introducing a PDDI system for cancer-screening accuracy assessment. We plan to conduct matching experiments based on actual data and compare them with the existing methods.

## Introduction

### Distributed Data Integration in Epidemiological Studies

With advances in information technology and enhanced data-collection systems, health databases are becoming increasingly abundant. Similar to other countries, the government and academic societies in Japan collect and manage a disease database. In addition, there are patient-based disease databases and population-based cohort study databases that are collected and managed mainly by research institutes [1-5]. Integrating health information held in these independent databases benefits epidemiological studies and public health practices; for example, it is possible to determine important correlations and causal relationships, such as between the onset of disease and the health status of an individual, which cannot be determined using a single database. Therefore, it is important to link databases managed by different institutions [6-8].

There are challenges associated with linking independent databases. The first is the guarantee of information privacy, including the handling of personally identifiable information. Concerns and considerations regarding privacy and data security are paramount; policies and regulations on the collection, use, and movement of personally identifiable information are becoming more stringent [9]. Therefore, in data linkage, sufficient measures to prevent the leakage of personal information are required, which have led to an increase in attendant costs, including labor. The second challenge is the construction of an efficient data linkage system. In countries where a unique identification key, such as the national identification number, is given to each individual and multiple medical or welfare-related data systems are linked, more efficient matching is possible compared with countries where such unique identifiers are not provided to every citizen. Nordic countries are representative of those using such unique identifiers. However, owing to privacy concerns, many issues need to be resolved before linking the databases; therefore, only a few countries have introduced such identifiers so far [10,11]. In countries where the unique identification key system has not been put into practical use, it is even more difficult to build a system that meets information privacy requirements and linkage efficiency. Consequently, it has been impossible to link databases managed by different institutions at a practical level in Japan.

### Secure Data Integration

To safely and effectively collate the data held by each institution in a decentralized state and use them, it is desirable to exchange only necessary information as much as possible without leaking personal information to the outside. However, without a unique identification key, it is common to use personal information, such as name and date of birth, as the key to perform matching [9,12]. The methods that are widely practiced today include one in which a data provider or user performs a matching operation or the method in which a data set containing personal information is passed to a third party (data depository) to perform the matching. Both methods require the movement of personal information that serves as the key to carry out the match. Although some studies [13,14] related to the linkage between 2 databases have been conducted, they are still vulnerable in terms of security and privacy. In fact, in a report by Kho et al [13], a hash value of names was used to match names so that a dictionary attack can determine which hospital a patient is in. A dictionary attack is a method in which the hash values of a precreated patient list are matched with the hash values stored in a system database. As the hash values of a limited range of data, such as patient lists, are vulnerable to a dictionary attack, the use of simple hash tables should be avoided. Furthermore, the proposal by Kho et al assumes that the database is owned by a single institution. In a report by Godlove et al [14], the system and other details were not described; therefore, the method of matching is a black box.

Therefore, strict countermeasures against information leakage and the costs involved are obstacles to conducting large-scale epidemiological studies. There are technical efforts to more securely approach a solution to this issue. Under the private set intersection protocol, which has been attracting attention in recent years, data other than those commonly included in data sets, distributed and managed by multiple data-holding institutions, are kept secret from other institutions; hence, only commonly included data are accessible [15-18]. The technology discussed in a previous report [18], which is an extension of private set intersection, focuses on the fact that a data set of medical-related information is generally composed of multiple attributes. After specifying an attribute as the matching key, the data associated with the same key attribute commonly included in each institution are integrated. It is called privacy-preserving distributed data integration (PDDI) because it integrates distributed data while ensuring privacy. Notably, unlike the proposal by Kho et al [13], PDDI does not simply match in the hash values of matching keys; therefore, information on whether a given patient is included in an institution is not available, and unlike Godlove et al [14], the specification is not a black box but is obvious. Studies on the application of newly developed PDDI systems to medical data are ongoing [19]. The PDDI system is expected to enable the secure integration of health information held in databases managed by different institutions and to enable epidemiological studies to be conducted with high security.

### Challenges in Implementing the Technology

PDDI is an established technology, but several additional steps must be taken before its implementation. The most important aspect is to show that the system can maintain sufficient matching accuracy and performance for operational purposes while keeping personal information secure, even when using actual data. The matching keys that are commonly used when a national identification number or similar identifier is not available, such as name and date of birth, include various errors, such as typing errors, at the time of input and orthographic variants owing to differences in the input format. Especially, in Japan, the lack of a standardized identification format also contributes to this effect. Therefore, the identification of identical persons tends to be associated with a certain rate of failure, lowering the matching accuracy [20]. Low matching accuracy affects outcome detection and narrows the research design and research themes to which the system can be applied. Matching accuracy is determined by the quantity and nature of such errors and the matching method [21,22]. The errors that can be found in data types used as matching keys are also affected by the language and characters used in the description. The optimal method for addressing these errors must be considered separately for different countries, regions, and databases. Various strategies have been developed to increase the reliability of matching. These include prior data cleaning, standardizing formats, combining personal information that serves as matching keys, and taking various measures such as probabilistic approaches [9,12,23,24]. However, it is unclear, especially in Japan, which data items can be used as matching keys to maximize the matching accuracy where a unique matching key cannot be used. The other aspect is the system performance. PDDI systems do not consolidate the data of each institution to 1 depository institution. The information held by each institution is encrypted within that institution, and the data are collected and distributed. However, the specifications of computer terminals of data-holding institutions and users vary considerably. Therefore, it is necessary to evaluate the performance of a linkage system for its stable use in a general-purpose environment.

The purpose of this project was to demonstrate that the security of personal information can be maintained in matching using actual data and that it is operationally accurate and performs significantly well for PDDI implementation and to identify which data items can be effective matching keys to perform data matching with high accuracy in situations where there is no unique matching key. However, because the use of personal information as a matching key is strictly controlled in Japan, a preliminary experiment was required using dummy data to experiment using actual data. In this study, we evaluated the protection of personal information, matching accuracy in cancer-screening accuracy assessment assuming a large-scale epidemiological study using artificially created data that simulate cancer screening and cancer registration data. If feasibility is confirmed in this study, we plan to carry out a verification study using actual data. The results of these studies are expected to be applied to large-scale population-based genomic cohort studies and large-scale studies using patient databases, thus contributing to further activation and development of database-based epidemiological research.

## Methods

### PDDI System

#### Overview

The features of PDDI used in this study are presented in our previous study [19], in which it is shown that PDDI consists of a secure computation server, data-holding institutions, and client. In PDDI systems, when there are multiple attributes per data sample, the database is divided into 3 types: key information, analysis target data, and *others*. The data to be analyzed, which are linked to the key commonly included in the database of each institution, are concealed and integrated. The key information and data to be analyzed may match. Important characteristics of PDDI systems are as follows:

1. No institution that uses the system, including those that own databases and those that receive data, can obtain any information other than the key information that is commonly shared between databases. Unlike the query-based method, the fact that 1 institution holds some information about the individual is not divulged to any other institution.
2. Key information used to match the data will not be divulged to any institution, including the PDDI secure computation server. In this paper, the PDDI secure computation server is denoted as PDDI server.
3. The processing time of each institution does not depend on the number of institutions involved in the system. There is no limit to the data available to each institution through the system.
4. No third-party institution collects or aggregates data to carry out matching.

**Figure 1:**
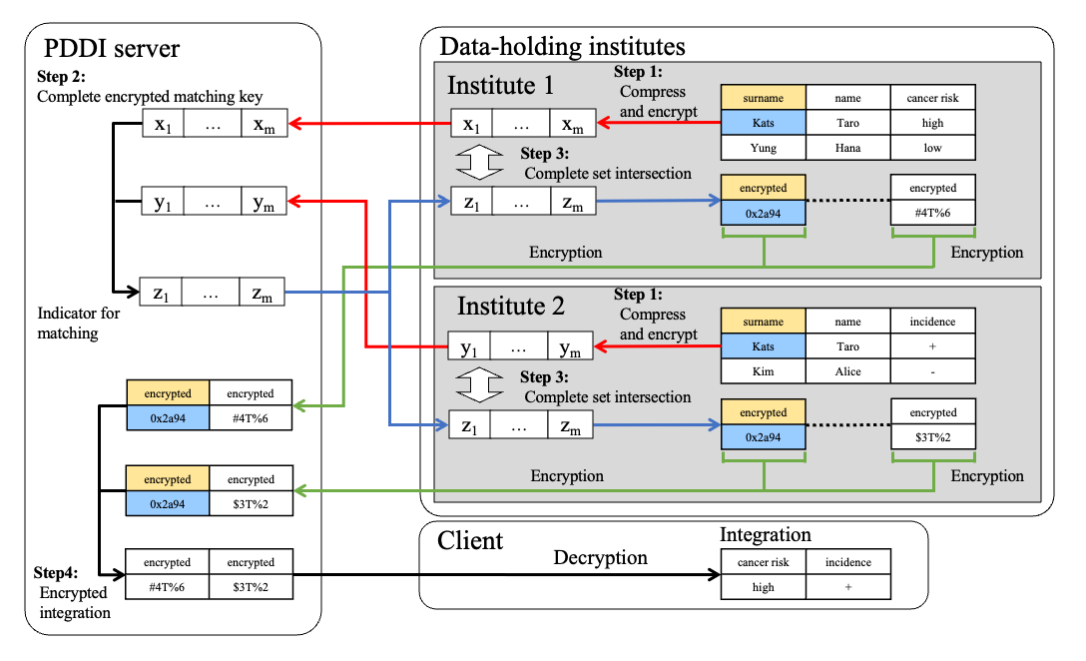
Schematic of the privacy-preserving distributed data integration (PDDI) system algorithm. Steps 1 to 4 represent each step of the merging process using the PDDI system described in the main text. The data held by each institution are encrypted and matched by the PDDI server using the data as the matching key. The analysis target data, which are related to the matching key without distinction between institutions, are decrypted only when they are provided to the client, and the matching-key information is never provided to the client.

We have described the PDDI algorithm in subsequent sections. Figure 1 shows the entire algorithmic process.

#### Step 1: Irreversible Compression and Encryption

Each institution compresses the key used for collating the data set with a hash function, converts it into unique and irreversible information, and sends the data encrypted by homomorphic and probabilistic encryption to the PDDI server.

#### Step 2: Creation of Matching Keys

The PDDI server calculates the sum of the encrypted data obtained from each institution (called an encrypted matching key) and sends these to each institution. Note that the PDDI server does not have the decryption key; therefore, it cannot decrypt the encrypted matching key.

#### Step 3: Analysis of Target Data for Set Intersection Computation

Each institution decrypts the received encrypted matching key and obtains the matching key used for extracting the key that is commonly included in all institutions. Next, the analysis target data related to the commonly included key are encrypted and sent to the PDDI server.

#### Step 4: Integration of Encrypted Analysis Target Data

The server integrates the encrypted analysis target data sent from each institution and sends it to the client; the matching-key information is not sent to the client. In this study, 1 data-holding institution evaluates whether the matching was performed correctly; therefore, the data-holding institution acts as a client.

These matching keys are transformed into Bloom filters and then encrypted in each institution. The encryption is probabilistic, and thus, the same plaintext is encrypted into different values. Furthermore, it cannot be decrypted without the collaboration of all institutions. Then, they are sent to the PDDI server. Note that the encryption of the compressed matching key is probabilistic, which implies that the ciphertexts of the compressed matching keys are not equal even if the compressed matching keys are equal. Therefore, by using the ciphertext, anyone cannot guess whether a patient with the matching key is included in the institute, unlike the proposal by Kho et al [13]. For the same reason, the PDDI server neither reveals any information of the matching key in each institution nor guesses whether a patient with the matching key is included in the institute. This is a completely different privacy policy from that proposed by Kho et al [13].

The PDDI implementation environment, environment construction, and usability are described in Multimedia Appendix 1. The basic part of this system (code, encryption, and others) is currently being prepared for publication.

### Experiment Model: Accuracy Assessment of Cancer Screening

#### Overview

In this study, we adopted accuracy assessment of cancer screening as a model for the matching experiment. Cancer screening is a general term for cancer-screening programs for the general population, which are conducted to reduce the mortality rate owing to early detection of cancer (secondary prevention). It is implemented around the world, centered on programs that have been scientifically recognized to reduce mortality, such as breast, cervical, and colorectal cancers [25-27]. The examinee is evaluated for the risk of having cancer based on the test results of each program. Patients who are determined to be at high risk, that is, those who are highly suspected of having cancer, are encouraged to visit a medical institution. Assessing the accuracy of cancer risk detection and controlling the quality of screening, so that the number of overlooked cancers and useless tests is kept to a minimum, constitute the major roles of cancer-screening accuracy control. Data on whether a patient who was judged to be at high risk in a program had cancer within a certain period (often 1-2 years) are required to assess the accuracy of cancer screening.

The biggest challenge in assessing cancer-screening accuracy is the collection and matching of distributed data. In many cases, cancer incidence, which represents the outcome of screening, needs to be obtained by matching with another source independent of the cancer-screening database; for example, a cancer registration database. In Japan, cancer-screening data are managed in a distributed state by the municipalities that are the implementing bodies. Moreover, cancer registration data are managed in a distributed manner by prefectures. Therefore, to collect and collate these data on a large-scale national or regional basis is difficult. The data size to be handled are large, and when there are many target municipalities, a lot of cumbersome procedures, which are not always standardized by the municipalities, are required to obtain the data. The greater the number of municipalities involved, the greater the movement of privacy information and the higher the risk of leakage. Therefore, in Japan, such studies are only conducted sporadically, using limited data from a small number of municipalities [28,29].

This system is characterized by no restrictions on the number of participating institutions or the amount of data held by the institutions and is considered an effective means for solving this problem. This system makes it easy to match the risk assessment information of distributed cancer screening with the cancer incidence information of cancer registration, which is expected to enable large-scale cancer-screening accuracy assessment, which has not yet been possible. Therefore, we surmised that applying a PDDI system for the assessment of cancer-screening accuracy is possible and devised an experimental plan using this model.

In cancer-screening accuracy assessment, indicators such as sensitivity, specificity, and positive predictive value are mainly used. If cancer screening indicates that there is a strong suspicion of having cancer (high risk), it is considered positive. In Japan, it is recommended to visit a medical institution, so this result is often called a “requiring detailed examination.” The other judgments are negative. Whether the patient has cancer is evaluated by comparing cancer incidence information in cancer registration data for 1 to 2 years from the date of consultation with the screening result. In other words, if the cancer screen is positive (there is a strong suspicion that the patient has cancer) and the cancer is subsequently diagnosed, the sensitivity, specificity, and positive predictive value in the context of assessment of the accuracy of cancer screening are defined as Textbox 1.

**Definition of items related to the accuracy of cancer screening**

- Screening sensitivity=Proportion of patients with cancer who screen positive
- Screening specificity=Proportion of patients without cancer who screen negative
- Positive predictive value for screening=Proportion of cases giving positive screen results who are already patients

The accuracy of cancer screening is indicated by adding “screening” to distinguish it from the accuracy of matching, which will be described in the “study design” section.

#### Background of Practical Data-Matching Failures

In countries that do not have a national identification number, such as Japan, data are generally collated using personal information. In such an environment, the accuracy of matching is reduced owing to various errors that may appear in the data points used as matching keys. The sources of errors when using matching keys are careless mistakes, orthographic variance owing to changes in culture and institutions, and differences in notation. The matching-key information may also change: change of address because of moving and renaming because of marriage. The prevalence of errors varies depending on the format adopted by the data holder and ability of the input person. They are also heavily influenced by the language in which the data are written. Japanese is the de facto official language in Japan, where we live, and it is adopted as the default language in most systems and services in Japan. Many errors in Japanese registry data are due to language-specific problems. Details of the errors originating from Japanese language features are described in Multimedia Appendix 2.

#### Study Design

As mentioned in the Introduction section, the purpose of this project is to demonstrate the safety, accuracy, and performance of data matching using the PDDI system and to identify effective data items as matching keys. This study is the first step of the project. We used the PDDI system to perform a data set matching experiment between simulated cancer-screening and cancer registration data sets, in which the PDDI system was tasked with matching data belonging to the same individuals between the sets. Feasibility was evaluated based on data security, matching accuracy (sensitivity and specificity), and system performance.

In this experiment, we performed matching under multiple conditions using personal information, such as first and last names, phonetic spelling, date of birth, and address, and evaluated how much matching accuracy could be obtained by combining matching keys. Various matching algorithms were devised to prevent a decrease in sensitivity while maintaining specificity [9,12,23]. However, the purpose of this study was to evaluate the PDDI system, not the novel matching method, to improve the matching accuracy; therefore, these advanced matching algorithms were not considered. Methods for more accurate and practical matching will be considered in the next steps of this project. Instead, we estimated how much the matching accuracy would affect the estimation of cancer-screening accuracy. The feasibility of applying the model in this study was evaluated.

Unlike conventional systems that use a simple hash function to compress privacy information or that require a single server to collect and process all data, our system uses the latest security techniques. For example, all data through the network are encrypted, and decryption cannot be performed by a single institution but only by the cooperation of all distributed institutions, without centralizing the data. Therefore, it is important to verify that it can be implemented on a general-purpose computer rather than on a special server. We evaluated the performance of the system, the total data processing time, memory use, and network traffic required by PDDI. The PDDI server was introduced to reduce the processing time and amount of communication between data-holding institutions. In practice, the data processing time of data-holding institutions and the total data processing time required to collect the information contained in common is of critical importance.

#### Setting of the Matching Experiment

Four data sets were created to simulate cancer-screening and cancer registration data for 2 types of cancers: colorectal and breast cancers. First, using the web-based test-data generation service that is open to the public in Japan, we created pseudodata that included name, gender, date of birth, and address to serve as matching-key information [30-32]. This service automatically creates personal information, such as name, date of birth, address, and telephone number, from random combinations, which is common in Japan. By selecting the required information items and the desired amount of generated data, the user can obtain data that simulate nonexistent personal information. To account for the possibility that data generated by any particular service may contain certain tendencies or biases, we generated one-third of all the data points from each of the 3 separate services. Next, from the created pseudodata, 60 cases of colorectal cancer and 62 cases of breast cancer were selected as common data that can be matched. These were commonly included in both cancer-screening and cancer registration data sets. To make the simulated data resemble the actual data, we consulted the staff who had abundant experience in registry management and a physician who is an expert in epidemiological research, and the data were modified to include errors and orthographic variants that are often empirically recognized. Experience shows that the number of errors in the data set is expected to be <10%. Previous studies have reported that the number of errors and omissions in the data available for matching keys in disease registries and medical and administrative databases is approximately 15% or less [33-35]. However, the actual prevalence of errors is unknown, as changes in culture and society are expected to affect their occurrence rates. Therefore, to create data that would be more difficult to match, the data were rewritten to increase the number of errors to the extent that a data point would have errors in multiple items. Errors were made more prevalent in the colorectal cancer data set than in the breast cancer data set such that the colorectal cancer data set would be more difficult to match than the breast cancer data set. Subsequently, the remaining pseudodata were added, and finally, a pseudo–data set of 2000 colorectal cancer screenings, 17,866 colorectal cancers, 1048 breast cancer screenings, and 29,949 breast cancers was created. Pseudodata items other than matching keys included serial numbers and pseudoidentification numbers for each database in all data sets. The following pseudodata were randomly added to the colorectal cancer-screening data set: test date, test results, and risk assessment of fecal occult blood test, which is commonly used in Japan. The diagnosis name; International Classification of Diseases, Tenth Revision code; and date of diagnosis were added to the cancer registration data set. Pseudodata items other than these matching keys were only decorative and did not affect the matching experiment. Table 1 lists the errors and orthographic variants added to the data set. The examples of errors specific to Japanese in the data sets used in the experiments in this study are shown in Figure S1 in Multimedia Appendix 2.

**Table 1:** Errors and orthographic variants included in the data set.

Class, error type, and matching key				Number of data points, n (%)		
				Colorectal cancer (n=60)		Breast cancer (n=62)
**Data entry errors**						
	**Typing errors**					
		Name	3 (5)		1 (2)	
		Birth date	15 (25)		0 (0)	
		Address	6 (10)		2 (3)	
		Sex	5 (8)		0 (0)	
	**Kanji conversion errors**					
		Name	5 (8)		6 (10)	
		Address	2 (3)		0 (0)	
	**Misreading**					
		Name	10 (17)		8 (13)	
	**Missing letters**					
		Name	2 (3)		1 (2)	
	**Omission**					
		Address	4 (7)		0 (0)	
		Name	10 (17)		1 (2)	
**Orthographic variants**						
	**Variant kanji**					
		Name	7 (12)		4 (6)	
	**Format**					
		Address	5 (8)		15 (24)	
**Data change**						
	**Name change**					
		Name	2 (3)		1 (2)	
	**Alias**					
		Name	2 (3)		0 (0)	
	**Moving**					
		Address	2 (3)		8 (13)	
Unmatched on multiple keys				25 (42)		14 (23)
Total				51 (85)		36 (59)

In the experiment, 6 pieces of information—family name (kanji or kana), first name (kanji or kana), date of birth, and gender—were used. In this experiment, matching was performed by combining ≥2 images. In the case of colorectal cancer, 57 combinations were possible: _6_C_2_ + _6_C_3_ + _6_C_4_ + _6_C_5_ + _6_C_6_. For breast cancer, outside of a small number of exceptional cases, all screening targets were females, and thus, only 26 combinations were possible: _5_C_2_ + _5_C_3_ + _5_C_4_ + _5_C_5_.

In the PDDI protocol, a data array called a Bloom filter is encrypted element by element. More than 90% of the total execution time is spent on this encryption process. The encryption of an element of the data array is independent of that of other elements, and parallelization is easy. The multiprocessing module in Python Standard Library (version 3.9; Python Software Foundation) was used for this parallelization. The PC environment used in the experiment was as follows: central processing unit (CPU), Intel (R) Xeon (R) CPU E5-2690 v4@2.60GHz (28 cores), memory 48 GB. The programs of all the institutions were executed on 1 PC.

#### Evaluation

Items related to matching accuracy are referred to below with “matching” to distinguish them from the accuracy of cancer screening. To calculate the matching accuracy, the pseudo–cancer screening data were used as a reference point, and when the data matched the specified matching-key conditions in the pseudocancer registration data, the match was considered *positive*. The case in which no matching data were present was defined as *negative*. This matching experiment was conducted between data sets in which the same persons were simulated in both data sets in advance. Therefore, the trueness and falseness of matching were determined as follows: cases in which the matching result correctly matched data belonging to the same person were considered *true* and those in which the matching result did not correctly match data belonging to the same person were considered *false*. In other words, a *false positive* means that data originally registered under separate individuals were erroneously matched, and a *false negative* means that data that should have been matched (because they belong to the same person) were not matched. In an environment in which matching keys that uniquely identify an individual are completely error-free, matching is perfectly accurate. In this experiment, as an evaluation of matching accuracy, the correspondence between positive and negative matches and their trueness or falseness was cross-tabulated to calculate the matching sensitivity and matching specificity. On the basis of this, a combination of matching keys with high matching sensitivity and matching specificity, that is, good matching accuracy, was extracted.

For the estimation of the effect of matching accuracy on the assessment of cancer-screening accuracy, we referred to past studies and assumed 2 scenarios: one in which the true accuracy of cancer screening involved a sensitivity of 90% and a specificity of 90% and the other with a sensitivity of 60% and a specificity of 90% [36-38]. Errors between true and estimated values were calculated to assess screening sensitivity, screening specificity, and screening positive predictive value. For matching accuracy, simulations were carried out in the following manner: values were changed in a stepwise manner in scenarios in which the matching sensitivity was 100%, the matching specificity was 100%, and each parameter was equivalent to the corresponding value observed in the matching experiment. The estimation assumed a group that underwent cancer screening in a certain year. The prevalence of new cancer incidence was set at 775.7 of 100,000 person-years based on the average prevalence in Japan. The data size did not affect the estimation, but at the time of calculation, it was set to 1000 people according to the parameters of this experiment.

In the performance evaluation experiment, we attempted to simulate a scenario in which the system is used by the institutions that are geographically distant from one another. Therefore, we used 6 computers installed at Osaka University and Yamaguchi University (4 of which simulated data-holding institutions). In the experiment, we measured CPU use, memory use, and network traffic for 3 data sizes: 2^10^, 2^12^, and 2^14^. We also implemented multiprocess parallelization and measured its speedup ratio.

### Ethics Approval

This study was approved by the institutional review board of the Kanagawa Cancer Center (2021 epidemiology-135).

## Results

### Data Protection

In our experiments, 2 distributed institutes independently held cancer screening and cancer registration data, in which each data set included the terms of birth date, first name, family name, and sex. These terms were used for matching keys. In our system, in addition to the use of probabilistic encryption, all matching keys and information through a network outside the institute are encrypted, and no server deals with raw data were stored in different distributed institutes. In other words, no institute has a decryption key and reveals all information. This implies that our system does not move any privacy information from any institute and thus avoids privacy risk.

### Matching Accuracy

**Figure 2:**
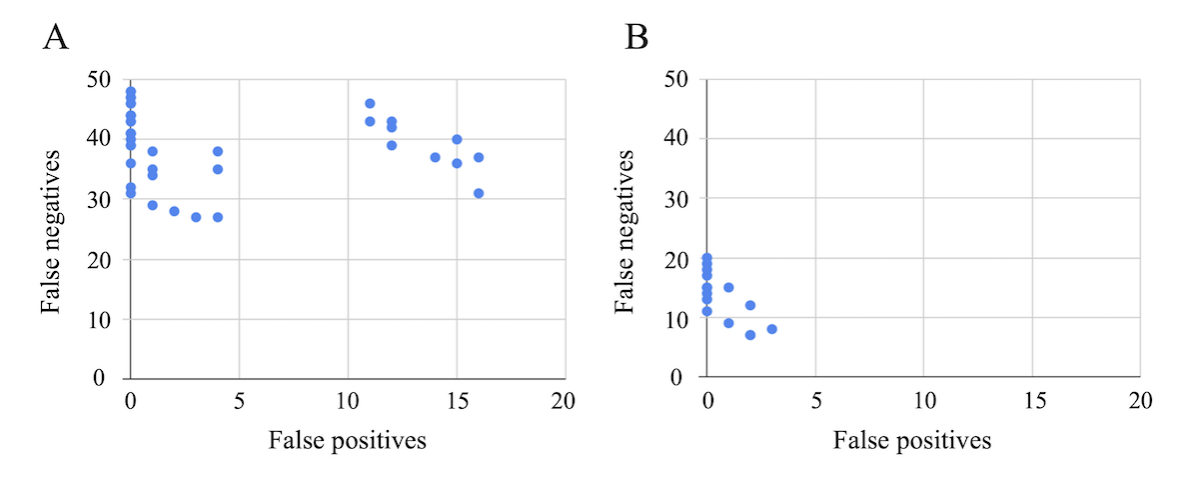
Number of false positives and false negatives. The points are placed according to the number of false positives and false negatives by the setting of each experiment conducted. Part A shows the result of data simulating colorectal cancer and Part B shows the result of data simulating breast cancer.

The results of matching using PDDI are shown in subsequent sections. From the preliminary experiments, when only 1 matching key is used, the number of false positives for matching increases and the specificity decreases significantly (Table S2 in Multimedia Appendix 3). Figure 2 shows the results of false positives and false negatives in which pseudodata of colorectal cancer and breast cancer were matched using various combinations of information. In the case of colorectal cancer data, the minimum number of false negatives for matching was 27 and the minimum number of false positives for matching was 0. It is desirable that the common data for all 60 items be output. However, up to 33 (60 – 27) cases are output correctly. For breast cancer data, the minimum number of false negatives for matching was 7, and the minimum number of false positives for matching was 0. Similarly, it is desirable that 62 common data items are output but a maximum of 55 (62 – 7) cases were output correctly.

Table 2 presents an excerpt of the matching results. Only combinations with a specificity of ≥99% are shown. In this pseudo–data set, it can be inferred that the combination of matching keys, including the date of birth, is particularly effective. In the colorectal cancer pseudodata, the combination with a specificity of ≥99%, the highest matching sensitivity was the one that used the date of birth and first name (kana) as keys; the matching sensitivity was 55.00%, and the matching specificity was 99.85%. For breast cancer pseudodata, the highest matching sensitivity was obtained when the date of birth and family name (kana or kanji) were used as keys: the matching sensitivity was 88.71%, and the matching specificity was 99.80%. In combination with 100% matching specificity, the matching sensitivity was 48.33% for the data simulating colorectal cancer and 82.26% for the data simulating breast cancer.

**Table 2:** Matching result between cancer-screening and cancer-registration data (excerpt).

Class^a^ and matching key		False positive, n	False negative, n	Sensitivity (%)	Specificity (%)
**Colorectal cancer**					
	Birth date, first name (kana)	3	27	55.00	99.85
	Birth date, first name (kana), family name (kana)	0	31	48.33	100
	Birth date, sex, first name (kana)	2	28	53.33	99.90
	Birth date, sex, family name (kana)	1	29	51.67	99.95
**Breast cancer**					
	Birth date, family name (kana)	2	7	88.71	99.80
	Birth date, family name (kanji)	2	7	88.71	99.80
	Birth date, first name (kanji)	1	9	85.48	99.90
	Birth date, first name (kana), family name (kanji)	0	11	82.26	100

^a^Results of the matching experiment between cancer-screening and cancer registration data for each matching key used. Cases in which all key data shown in the matching-key column successfully corresponded were considered positive matches.

Table 3 shows the effect of matching accuracy on the estimation of sensitivity and specificity of cancer screening based on the model used in this experiment, an assessment of the accuracy of cancer screening. The matching sensitivities were approximately 85%, 50%, and 90%, and the matching specificities were 99.9%, 99.8%, and 99.99%. Assuming that the original values of both screening sensitivity and specificity are both 90% if the matching specificity is set to 100% and the matching sensitivity values are reduced to 90%, 85%, and 50%, the apparent screening specificity values become 89.94% (−0.06%), 89.91% (−0.10%), and 89.69% (−0.34%), respectively. Thus, as the matching sensitivity decreases, the screening specificity is underestimated. If the matching specificity decreases, the screening sensitivity is underestimated. On the basis of the experimental results of the data set simulating breast cancer, when calculated with a matching sensitivity of 88.71% and matching specificity of 99.80%, the apparent value of the screening sensitivity was 72.09% (−19.9%) and that of the screening specificity was 89.93% (−0.08%), and the rate of change in the apparent value of the screening sensitivity was large. However, when using the results of another combination and calculating with a matching sensitivity of 82.26% and matching specificity of 100%, the apparent value of screening sensitivity is 90% (no decrease), and the apparent value of screening specificity is 89.89% (−0.12%). In other words, when the matching specificity is sufficiently large, even if the matching sensitivity is a little low, the change from the original value for both screening sensitivity and screening specificity remains small. As shown in Table 3, this tendency was maintained, even in the estimation assuming the original screening sensitivity of 60%. In addition, regarding the positive predictive value of screening, a decrease in matching sensitivity makes the positive predictive value of screening appear smaller than the original value, and a decrease in matching specificity makes the positive predictive value of screening appear larger than the original value. The effect of matching specificity is also greater for the positive predictive value of screening.

**Table 3:** Estimation of the impact of matching accuracy on the screening accuracy^a^.

Assumption of matching accuracy (%)			Screening sensitivity (%)			Screening specificity (%)			Positive predictive value (%)	
Sensitivity	Specificity	True		Estimate	True		Estimate	True		Estimate
90	100	90		NA^b^	90		89.94	6.6		5.92
85	100	90		NA	90		89.91	6.6		5.59
50	100	90		NA	90		89.69	6.6		3.29
100	99.99	90		88.99	90		NA	6.6		6.58
100	99.90	90		80.93	90		NA	6.6		6.67
100	99.80	90		73.70	90		NA	6.6		6.76
*88.71*	*99.80*	*90*		*90.00*	*90*		*89.89*	*6.6*		*6.02*
*82.26*	*100*	*90*		*72.09*	*90*		*89.93*	*6.6*		*5.41*
90	100	60		NA	90		89.96	4.5		4.03
85	100	60		NA	90		89.94	4.5		3.81
50	100	60		NA	90		89.81	4.5		2.24
100	99.99	60		59.37	90		NA	4.5		4.49
100	99.90	60		54.33	90		NA	4.5		4.58
100	99.80	60		49.81	90		NA	4.5		4.67
*88.71*	*99.80*	*60*		*48.81*	*90*		*89.96*	*4.5*		*4.17*
*82.26*	*100*	*60*		*60.00*	*90*		*89.68*	*4.5*		*3.18*

^a^The table shows the impact of matching accuracy on cancer-screening accuracy estimates when the true sensitivity of cancer screening is set at 90% and 60%, and the true specificity is set at 90%. The cancer incidence rate is approximately 775.7 person per year, which is the national average in Japan.

^b^NA: not affected. “NA” represents that no change occurred between the true and estimated values. The italicized values show the estimates obtained using the experimental data.

In principle, when the matching sensitivity is 100%, even if the matching specificity is reduced, both true-negative and false-positive cancer screenings are misidentified as having cancer at the same rate. Therefore, the specificity of cancer screening does not change. Similarly, when the matching specificity is 100%, even if the matching sensitivity decreases, both true-positive and false-negative cancer screening will be misidentified as “no cancer” at the same rate. Therefore, the sensitivity of cancer screening does not change. Therefore, these values are not shown and are depicted as not affected, except when the matching sensitivity and matching specificity obtained from the matching experiment are used.

### Performance

**Figure 3:**
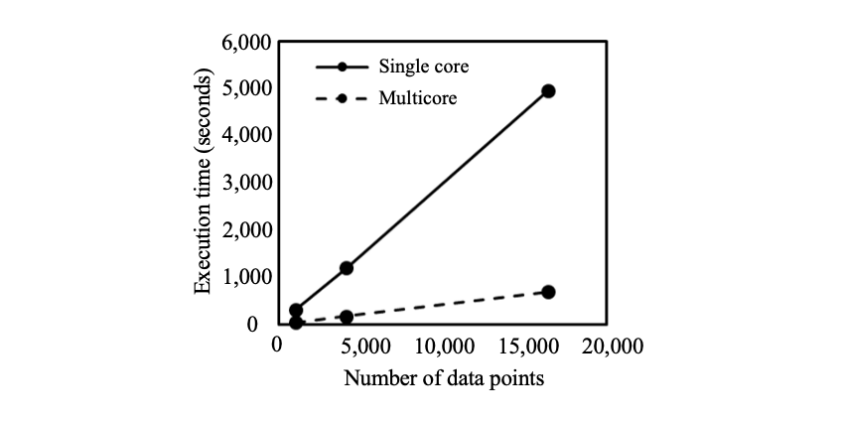
Execution time. The graph shows the relationship between the amount of data and the execution time. The solid line shows the execution time without parallelization, and the dashed line shows the execution time with parallelization.

The results of the performance evaluation experiment are in subsequent sections. The specifications of the computer used in the experiment are listed in Table S1 in Multimedia Appendix 1. Figure 3 shows the relationship between the amount of data and execution time.

**Figure 4:**
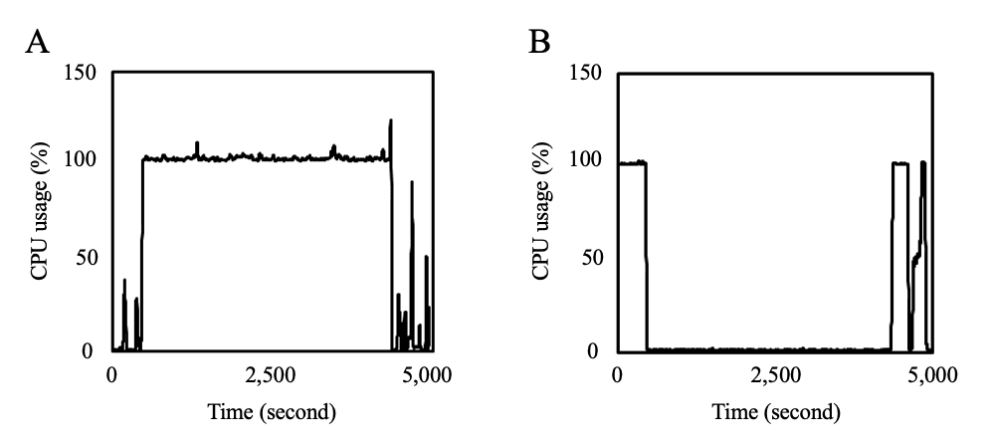
Changes in central processing unit (CPU) usage. The graphs show the changes in CPU usage of the privacy-preserving distributed data integration (PDDI) server and the data-holding institutions when the process is executed on 214 datapoints without parallelization. Part A represents the results of the PDDI server, and part B represents the results of the data-holding institution.

As shown in Figure 3, the amount of data and the execution time are almost proportional. Furthermore, with 2^14^ (16,384) data points, the nonparallelized execution time was 82 minutes and 26 seconds, whereas with parallelization, the execution time was 11 minutes 38 seconds; hence, a 7.1-fold speedup was observed with parallelization. Figure 4 shows the changes in CPU use of the PDDI server and data-holding institutions when the process is executed on 2^14^ data points without parallelization. As can be observed in this graph, 80.67% of the execution time is processed by the PDDI server, and the calculation time of the data-holding institutions is only 19.33%.

**Figure 5:**
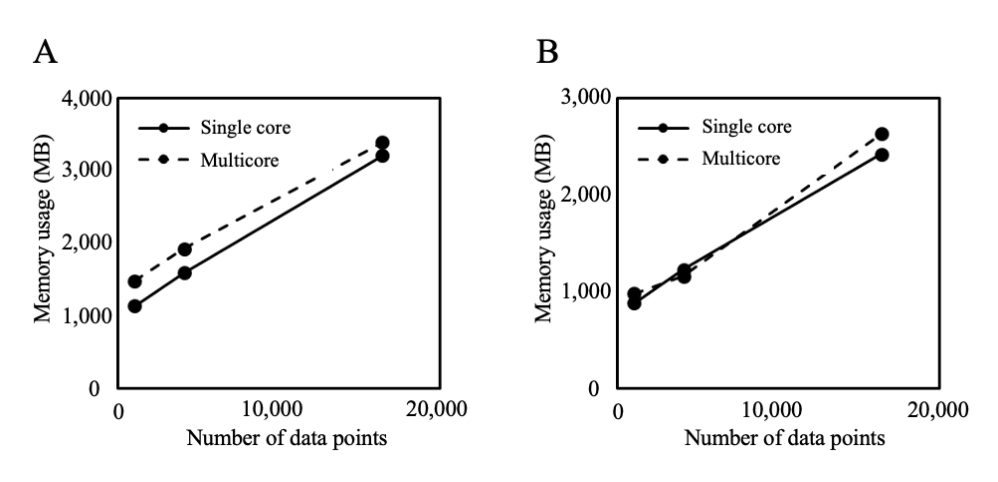
Memory usage. The graphs show the relationship between the amount of data and the memory usage of the privacy-preserving distributed data integration (PDDI) server and the data-holding institutions. Part A represents the results of the PDDI server, and part B represents the results of the data-holding institution.

Figure 5 shows the relationship between the amount of data and memory use of the PDDI server and data-holding institutions. Memory use increases linearly with the amount of data. However, even during parallelization for 2^14^ data, which uses a large amount of memory, the PDDI server required no more than 3.4 GB of memory, and the data-holding institutions required no more than 2.7 GB of memory.

## Discussion

### Evaluation of Matching Experiment

In this study, we conducted a matching experiment using the accuracy assessment of cancer screening as a model by matching the cancer-screening and cancer registration data.

In the experiment, any matching information is transformed into Bloom filters, encrypted within each institution, and then sent to the PDDI server. Probabilistic encryption was used in this study. This implies that the same matching key is compressed and randomly encrypted to different ciphertext, for example, each birth date of patients A and B in cancer registration data set is 19970911, but that compressed and randomly encrypted are not equal to each other. Unlike simple matching using a hash value [13], our scheme is secure against dictionary attacks because the same value is encrypted into different values owing to the probabilistic encryption.

The matching keys used for multiple combinations, which were particularly excellent with few false positives and false negatives, were all registered in most databases in Japan. It is highly likely that these keys can be applied to existing databases. The matching sensitivity remained in the 50% range for simulated colorectal cancer data containing 85% matching-key errors, but in the case of simulated breast cancer data, which contained 59% matching-key errors, the matching sensitivity value was approximately 85%. This experiment was conducted in a manner that intentionally created a data set that was difficult to match owing to a high prevalence of errors and a large amount of data containing errors in multiple matching keys. The errors contained in the 2 data sets differ as shown in Table 1, and these results cannot be simply compared, but, in general, the fewer the number of errors in the matching keys, the better the matching accuracy. Although cultural backgrounds and times vary, previous studies have shown that the number of errors and omissions in disease registries, medical, and government databases is <15% for matching-key data such as name, zip code, and date of birth [33-35]. On the basis of the opinions of staff with abundant experience in registry management, we predicted that up to approximately 10% of the actual data used for cancer-screening accuracy assessment in Japan includes an error in the matching key. In principle, the false-negative rate cannot be greater than the percentage of data with errors contained in the data set; therefore, it is estimated that a matching sensitivity of ≥90% can be obtained in verification experiments using actual data. The error distributions of the 2 data sets in this experiment were the same, and the prevalence was set at 10%. In the colorectal cancer data, the matching sensitivity was 94.70% when the date of birth and first name (kana) were used as the matching key. In breast cancer data, the matching sensitivity was 98.09% when the date of birth and family name (kana or kanji) were used as the matching key. Regarding the specificity of matching, the combination of keys shown in Table 2 maintained a high specificity of ≥99% in this estimation.

In practical use, the influence on the outcome and evaluation index to be obtained by performing matching is more important than the numerical value of the matching accuracy. As shown in Table 3, when assessing test accuracy for infrequent events, such as cancer, changes in matching specificity values have a significant effect on the apparent value of test accuracy. In our model, a slight decrease in matching sensitivity had a relatively small effect on screening sensitivity and screening specificity. In other words, it is highly important to keep the matching specificity as high as possible to prevent underestimation of the screening sensitivity and screening specificity. The estimation shows that a combination of matching keys with 100% matching specificity has a small effect on the sensitivity and specificity of cancer screening, even if the matching sensitivity is low. Assuming that the original screening sensitivity and screening specificity are 90%, even when the matching specificity is not 100% if the matching specificity is ≥99.97%, the screening sensitivity maintains within 5% even if the matching sensitivity is 85%. Therefore, when considering the accurate calculation of sensitivity estimates for cancer screening, it is desirable to select a matching-key or matching algorithm that can improve matching sensitivity as much as possible without reducing matching specificity. Matching specificity has a greater effect than matching sensitivity on the positive predictive value of screening. However, it is more susceptible to matching sensitivity than screening sensitivity or screening specificity. Therefore, when focusing on the positive predictive value of screening as the index, it is necessary to select the matching key in consideration to not only the matching specificity but also the decrease in matching sensitivity.

Matching specificity in this experiment is defined as the value obtained by dividing the number of people who are determined not to have cancer as a result of matching by the number of people who do not have cancer among the data included in the cancer-screening data set. Therefore, the specificity of the match is affected by the ratio of the data size of the cancer registration data set to the cancer-screening data set and the percentage of true patients with cancer included in the cancer-screening data set. The cancer-screening and cancer registration data sets used in this experiment were approximately 1000 to 2000 and approximately 17,000 to 30,000, respectively. In Japan, where the cancer-screening rate is low, this is roughly equivalent to the number of cancer screenings in small municipalities and the number of cancers in large prefectures; cancer-screening data are managed for each municipality that is the implementing body, and cancer registration data are managed by each prefecture. Epidemiological studies may have to deal with even larger cancer-screening data. In this case, the difference in data size from the cancer registration data set is smaller than that in this experiment. Therefore, matching specificity is expected to be higher. As the errors of the data set in this experiment do not necessarily reflect the actual prevalence, the sensitivity and specificity in this experiment are just reference values. Even so, it is expected that the PDDI system can be used for the assessment of cancer-screening accuracy using matching with cancer registration data by appropriately adjusting the matching conditions.

Performance evaluation experiments verified that the execution time of the PDDI system was almost proportional to the amount of data, and the execution time in parallel execution was 43 seconds per 1000 data samples. With the pseudodatabase used, the execution was completed in approximately 21 minutes, which is sufficient performance for epidemiological studies. The effect of the performance of the computer installed in the data-holding organization on the execution time is relatively small, approximately 20% of the total, and the memory use is <1 GB. Therefore, it was proven that the processing speed was acceptable even with the performance of a normal laptop PC. The maximum network traffic of the PDDI system in this experiment was 858 Mbps. Even so, the execution time consumed by communication is small, and if the communication speed of the data-holding organization is ≥10 Mbps, we do not believe that there will be any problems using this system.

### Challenges for Next Experiments Using Practical Data

On the basis of this study, we plan to conduct a verification experiment using actual cancer-screening and cancer registration data. In this experiment, the number of errors in the actual data were unknown. Therefore, the experiment was conducted using a data set with a large number of errors. In the next matching experiment using actual data, we plan to determine the degree of matching accuracy that can be obtained in comparison to a method that partly uses matching based on human judgment. On the basis of this, it is possible to realistically estimate the extent to which matching can cause errors in examination accuracy. Therefore, it is possible to perform higher quality evaluations for practical use. Regarding performance evaluation, as shown in the results of this experiment, the calculation time and memory consumption of the terminal depend on the amount of data. The main purpose of this experiment was to evaluate the feasibility, and the data set used was with a smaller number of items than those contained in the actual data. Therefore, in the next stage, we will confirm the performance using data on the scale of municipalities and prefectures that may actually be used. On the basis of these results, it is necessary to perform a trial calculation to determine the size of the data set that can be matched.

### Implementation for Practical Epidemiological Studies

Through this experiment and estimation, we demonstrated that the use of matching using the PDDI system for cancer-screening accuracy assessment deserves consideration. This system is expected to be applied to other types of epidemiological research because it assists in data matching between databases managed by different institutions. We considered the applicability based on matching sensitivity and specificity using cohort studies and case-control studies, which are typical epidemiological studies, as examples.

Assuming that a cohort study examining the association between a factor and cancer incidence will determine the risk ratio of cancer incidence with people who have the factor compared with those who do not have, each person’s data in the cohort are matched with cancer registration data to record cancer incidence. The estimation of this setting is presented in Table S3 in Multimedia Appendix 4. The risk ratio does not change from the true value only by the decrease in matching sensitivity. If the matching specificity is reduced, the risk ratio is underestimated. However, it can be seen from the estimation that the decrease in the risk ratio is approximately 10% in the matching sensitivity and matching specificity equivalent to this matching experiment, even when the prevalence of the factor is 75%. Next, let us assume a case-control study using a data set that links the factors to be examined with data on the presence or absence of a disease by matching. Table S4 in Multimedia Appendix 4 shows a common disease with a high prevalence, here a trial calculation for diabetes, and Table S5 in Multimedia Appendix 4 shows a trial calculation for ulcerative colitis as an example of a disease with a low prevalence. Poor matching accuracy causes systematic errors in factor exposure in populations and control populations, which tends to underestimate odds ratio estimates. Occasionally, this has a greater effect on the odds ratios in diseases with low prevalence. Therefore, when assuming the use of the PDDI system in cohort and case-control studies, care must be taken in selecting the target disease and underestimating the odds ratio. However, if appropriate calculations are made, it appears that a large variety of applications can be fully examined.

The advantage of the PDDI system is that it can provide data to users in an already-matched state, even among ≥3 databases. Currently, in research that integrates data managed by different institutions without a unique identification key, a step-by-step process is necessary, such as collecting data from all target institutions and then performing a match or narrowing down the target audience and repeating the match. However, in the PDDI system, although the data are distributed and stored in different institutions, it is possible to retrieve matched data that meet these conditions. As in other methods [39], it does not assume prior linkage. Therefore, the PDDI system is particularly useful when data obtained from the databases of ≥3 institutions are combined and analyzed. Owing to this characteristic, this system enables the safe and efficient integration of data even in an environment such as Japan, that is, an environment where cancer-screening data are distributed and stored in many municipalities and, therefore, requires multiple movements of private information.

### Limitations

This study has several limitations. This study was conducted as a preliminary step in the experiments using real-life data. The data set used in this experiment is a pseudo–data set created using software that is open to the public and does not reflect the amount or ratio of errors mixed in the actual data, nor does it cover all types of errors contained in real-world data. As the types and number of errors contained in actual data depend on the input style of each database and the ability of the input person, subsequent verification experiments using actual data are required. In this study, we dealt only with matching under the condition that all the selected matching keys matched and did not use complicated algorithms for partial matches. We did not examine the extent to which the matching sensitivity and matching specificity shown in this study can be improved by further improvements in matching methods. The experiment used a local database in Japan as the environment, and we noted that the error format is also influenced by language, culture, and institution. Therefore, it is unlikely that this result can be applied directly to other countries and regions.

### Conclusions

As a first step toward implementing PDDI in epidemiological studies, we evaluated its feasibility in a model of cancer-screening accuracy assessment in terms of safety, matching accuracy, and performance through a matching experiment using dummy data. This system makes it possible to collate only the information related to the shared data without disclosing the data distributed and managed by multiple institutions and without using a third party. In the matching experiment and the estimation of the effect on the cancer-screening accuracy index using the matching sensitivity and matching specificity obtained by the experiment, it was shown that screening sensitivity and screening specificity can be assessed with minimal errors by keeping the matching specificity high. Because of its characteristics, this system reduces the labor and costs required for personal information management and collation work for both researchers and data providers in many epidemiological studies and is expected to further improve the efficiency and speed of research activities. In future, we will carry out further verification for practical use by using existing data and comparing it with existing methods.

## Appendix

Multimedia Appendix 1 Privacy-preserving distributed data integration (PDDI) implementation environment, environment construction and usability.

Multimedia Appendix 2 Cultural background of practical data-matching failures and examples of the errors specific to Japanese in the dataset of the experiment.

Multimedia Appendix 3 Matching-key combinations and the matching results that were not described in the text.

Multimedia Appendix 4 Estimating the impact of matching accuracy on outcome evaluation in epidemiological studies.

## Abbreviations

CPU: central processing unit
PDDI: privacy-preserving distributed data integration
