# Supplementary material for: A Privacy-Preserving Distributed Medical Data Integration Security System for Accuracy Assessment of Cancer Screening: Development Study of Novel Data Integration System
Source: JMIR Med Inform. 2022 Dec 30;10(12):e38922. doi: 10.2196/38922 (PMC9840098; doi:10.2196/38922)
Supplement: Multimedia Appendix 1 [file medinform_v10i12e38922_app1.docx]

## Multimedia Appendix 1

**PDDI Implementation Environment and Environment Construction**

The design policy of PDDI software is as follows:

1. Implement only core business logic
2. Settings can be made only with the configuration file
3. Deployment can be done as automatically as possible
4. Communicate using Restful API to facilitate Web linkage
5. Communicate as collectively as possible

By implementing only the business logic, the institutions that use PDDI can customize it prior to use. For example, for non-PDDI mechanisms such as institutional certification, end-users will be able to use general certification software. We eased the hurdles of software deployment by allowing customization to occur simply by changing the configuration file. With our software, the matching key can easily be set, and so on. Because the experimental environment can be built using Docker, it is possible to deploy using Docker even in the production environment. Therefore, a few items are to be set at the time of deployment.

Restful API is defined for the interface part of network communication, and communication is performed via this API. This makes it easy to link with other web applications and debug when using the network. By sending and receiving data together as much as possible, the network performance is improved. This is implemented using Python language and is easy to maintain at the source-code level.

The method of constructing the experimental environment is described below. Because PDDI software uses Docker and Docker Compose, the environment for execution ends with only the following two commands:

$ docker-compose build

$ docker-compose up -d

By checking with the docker command after starting the container, the four containers operate as shown below.

$ docker ps

CONTAINER ID IMAGE

a59c5548665f docker_party2

2f7cb5081da1 docker_dealer

620438d4ecd1 docker_party1

8f067f27bd6f docker_client

Here, docker_party described in IMAGE is a data-holding institution, docker_dealer is a PDDI secure computation server, and docker_client is an organization that uses integrated data. After starting the container and attaching it to the container, PDDI operates.

For example, a data integration institution runs with docker_dealer, as follows:

$ docker attach pddi-dealer

root@dealer #> python3 /pddi/server.py

* Running on <http://0.0.0.0:5000/>

In this way, this software makes it very easy to execute the program.

**PDDI Usability**

The PDDI system aims to improve the usability and design of the user interface based on the following guidelines:

Intuitive operation: We assumed that the PDDI system users would likely be medical personnel, i.e., that they are not always familiar with information and communication technology (ICT technology). For this reason, it was designed so that almost all operations other than the initial settings can be completed with just a mouse click. Consideration was given to screening transitions that allow users to understand the flow of operations naturally without having to read the manual.

Prevention of errors: The PDDI system handles the data that require strict security, such as medical information. For this reason, usability restrictions have been set to prevent human errors such as accidentally disclosing personal information to other data-holding institutions. First, the data-holding agency's application cannot directly connect to other databases. The user of the data-holding organization creates new CSV format data and registers it in the application. The attributes used for matching and integration are not used in the protocol without explicit permission from the data-holding institution.

Elimination of complexity: The operating principles of the encryption protocol of this program are completely separated from the user interface, and the user does not need to be aware of what encryption protocol is used to realize the system. Therefore, changing the encryption protocol has no effect on the operating procedure. Only system administrators can access settings that require specialized knowledge, such as parameters used for encryption processing and hashing.

Ease of introduction: Scalability, which is one of the features of the PDDI system, that is, high extensibility against an increase in the number of institutions, is realized not only in the processing time but also in the introduction. Only the address of the PDDI secure computation server and the password issued by the administrator need to be given to the new data-holding institution, and no coordination with other data-holding institutions is required. In addition, the data matching method used in this system does not require a centralized data repository, and safety is guaranteed no matter where the PDDI secure computation server is installed.

| Table A1. Specifications of the computational devices used in this experiment | | | | |
| --- | --- | --- | --- | --- |
|  | CPU | Clock rate | Logic processors | Memory |
| PDDI secure computation server | Intel(R) Xeon(R) CPU E5-2620 v4 | 2.1 GHz | 16 | 32 GB |
| Data-holding institution 1 | Intel(R) Core(TM) i7-7700HQ CPU | 2.8 GHz | 8 | 12.8 GB |
| Data-holding institution 2 | AMD Ryzen 7 5800X 8-Core Processor | 3.8 GHz | 16 | 25 GB |
| Data-holding institution 3 | Intel(R) Xeon(R) CPU E5-2690 v4 | 2.6 GHz | 28 | 48 GB |
| Data-holding institution 4 | AMD Ryzen 9 5900X 12-Core Processor | 3.7 GHz | 24 | 130 GB |
| Client | AMD Ryzen 9 5900HS with Radeon Graphics | 3.0 GHz | 16 | 25.1 GB |
