# Supplementary material for: A Privacy-Preserving Distributed Medical Data Integration Security System for Accuracy Assessment of Cancer Screening: Development Study of Novel Data Integration System
Source: JMIR Med Inform. 2022 Dec 30;10(12):e38922. doi: 10.2196/38922 (PMC9840098; doi:10.2196/38922)
Supplement: Multimedia Appendix 2 [file medinform_v10i12e38922_app2.docx]

## Multimedia Appendix 2

**Cultural Background of Practical Data-Matching Failures**

Japanese is mainly written in three types of characters: kanji, hiragana, and katakana. Kanji are logograms derived from Chinese characters. Hiragana and katakana are collectively called "kana" and are syllabic characters that indicate pronunciation. They are mainly used to write words that do not use kanji or to indicate the pronunciation of kanji. Because there are many types of kanji, if one needs to input a specific kanji when typing Japanese, it is necessary to first enter the pronunciation of that character in kana and then select it from various possible candidates offered by the input software. This process is called the kanji conversion. In Japanese names, kanji is used in most family names and many first names. The pronunciation of kanji used in the first names is quite diverse because individuals have the freedom to independently determine the pronunciation of the name and the characters associated with it. Therefore, in many systems and services, the syllabic characters are recorded alongside the registered person's kanji name.

Given these linguistic characteristics, the sources of errors when using matching keys written in Japanese are careless mistakes (typing, kanji conversion, inputting kana corresponding to kanji, missing letters, and omissions), orthographic variance due to changes in culture and institutions (variant characters of kanji, old syllabic characters), and differences in notation (differences in format for each database, restrictions on the orthographic variants that can be used, and habits of input personnel). The matching-key information may also change owing to the use of alias by foreigners residing in Japan (a Japanese-style name can be registered as an alias in the residence certificate in addition to the real name).

The examples of errors specific to Japanese in the datasets used in the experiments in this study are shown in Figure A1.


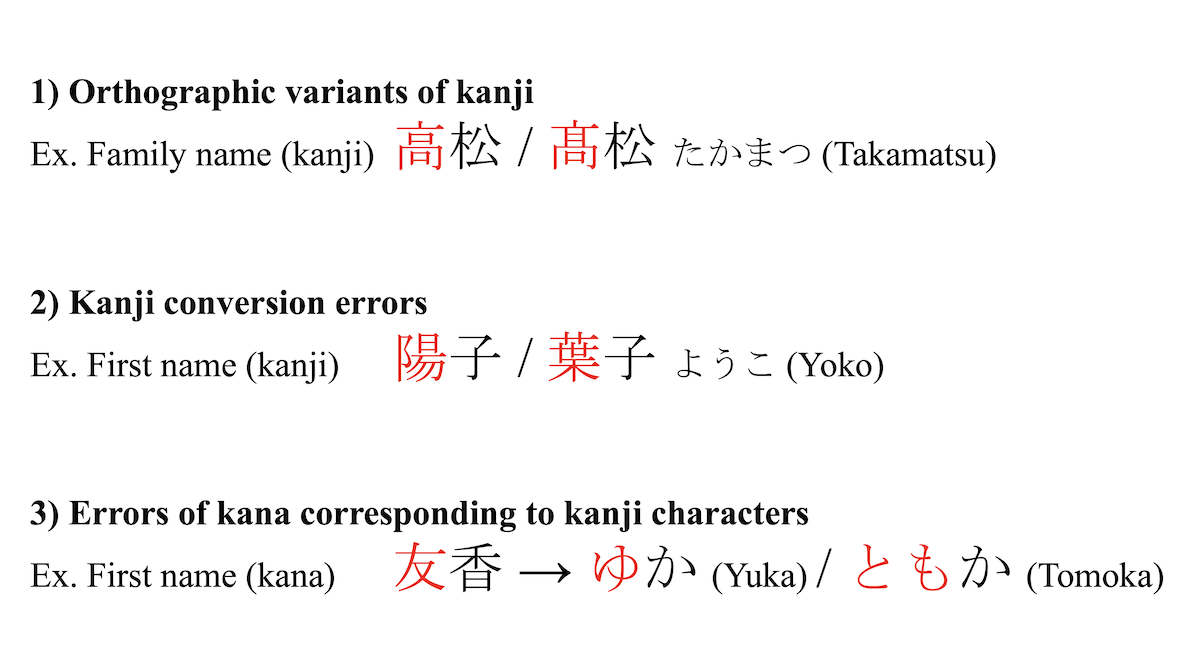


Figure A1. Examples of the errors specific to Japanese in the dataset of the experiment. 1) Orthographic variants of kanji may cause errors because kanji have many variants, especially those used in family names. If the original orthographic kanji variant used in the data is in the minority and an input method editor does not support it, a major variant may be input instead of the original one. 2) During kanji conversion, many kanji characters with the same kana are suggested as input candidates, which may cause errors. 3) If kanji or its combination has more than one major pronunciation or the original pronunciation is unusual, input errors in kana corresponding to the kanji may occur.
