# Supplementary material for: A Privacy-Preserving Distributed Medical Data Integration Security System for Accuracy Assessment of Cancer Screening: Development Study of Novel Data Integration System
Source: JMIR Med Inform. 2022 Dec 30;10(12):e38922. doi: 10.2196/38922 (PMC9840098; doi:10.2196/38922)
Supplement: Multimedia Appendix 3 [file medinform_v10i12e38922_app3.docx]

## Multimedia Appendix 3

| Table A2. Matching keys with large numbers of false-positive matches | | | | | |
| --- | --- | --- | --- | --- | --- |
| Cancer type | Matching keys | False positive | False negative | Sensitivity | Specificity |
| Colorectal cancer | Birthdate, sex | 370 | 21 | 65.0% | 80.9% |
|  | First name (kanji, kana) | 18,633 | 30 | 50.0% | NC |
|  | Sex, first name (kanji) | 19,136 | 26 | 56.7% | NC |
|  | Sex, family name (kanji) | 12,774 | 25 | 58.3% | NC |
|  | Sex, first name (kana) | 64,057 | 18 | 70.0% | NC |
|  | Sex, family name (kana) | 13,665 | 20 | 66.7% | NC |
|  | Sex, first name (kanji, kana) | 18,411 | 34 | 43.3% | NC |
|  | Sex, family name (kanji, kana) | 11,014 | 32 | 46.7% | NC |
| Breast cancer | Family name (kana),  first name (kana) | 309 | 13 | 79.0% | 68.7% |
|  | Family name (kanji, kana) | 29,844 | 12 | 80.6% | NC |
|  | Family name (kanji),  first name (kana) | 314 | 11 | 82.3% | 68.2% |
|  | Family name (kanji),  first name (kanji) | 72 | 14 | 77.4% | 92.7% |
|  | Family name (kana),  first name (kanji) | 95 | 15 | 75.8% | 90.4% |
|  | First name (kanji, kana) | 42,306 | 15 | 75.8% | NC |
|  | Family name (kanji, kana),  first name (kana) | 212 | 15 | 75.8% | 78.5% |
|  | Family name (kana),  first name (kanji, kana) | 91 | 19 | 69.4% | 90.8% |
|  | Family name (kanji, kana),  first name (kanji) | 71 | 18 | 71.0% | 92.8% |
|  | Family name (kanji),  first name (kanji, kana) | 68 | 17 | 72.6% | 93.1% |
|  | Family name (kanji, kana),  first name (kanji, kana) | 68 | 20 | 67.7% | 93.1% |
| NC = not calculated | | | | | |

The table lists the matching-key combinations and the matching results that were not described in the text because there were many false positives in the matching experiment. Of the 2,000 colorectal-cancer screening data, 60 had cancer, and of the 1,048 breast-cancer screening data, 62 had cancer. Those who were judged to have a strong suspicion of having cancer by cancer screening were defined as positive for cancer screening, and those who were not were defined as negative. We determined the trueness or falseness of the match by evaluating its correspondence with the true cancer incidence.

In this experiment, cancer-screening data was set as the reference point, and data from the cancer-registration dataset were matched according to their correspondence in terms of predetermined matching keys. In this method, if the data specified in the matching key are not diverse, multiple data points in the cancer registration dataset can match. The number of false positives in Table A2 is counted in reference to the screening dataset: multiple matches to a single datapoint are still counted as one false positive. Because the specificity in such a case was not calculated, it is described as not calculated (NC).
