# Supplementary material for: A Privacy-Preserving Distributed Medical Data Integration Security System for Accuracy Assessment of Cancer Screening: Development Study of Novel Data Integration System
Source: JMIR Med Inform. 2022 Dec 30;10(12):e38922. doi: 10.2196/38922 (PMC9840098; doi:10.2196/38922)
Supplement: Multimedia Appendix 4 [file medinform_v10i12e38922_app4.docx]

## Multimedia appendix 4

| Table A3. The effect of matching accuracy on the estimation of the risk ratio given by a factor in a cohort study | | | | | |
| --- | --- | --- | --- | --- | --- |
| Prevalence of factor | Assumptions of  matching accuracy | | Number of incidences | Risk ratio | |
|  | Sensitivity | Specificity |  | True | Estimate |
|  | 5% | 100% |  |  | 1.500 |
|  | 100% | 99.99% |  |  | 1.492 |
| 75% | 100% | 99.90% | 6,347 | 1.500 | 1.425 |
|  | 100% | 99.80% |  |  | 1.369 |
|  | 88.71% | 99.80% |  |  | 1.357 |
|  | 82.26% | 100% |  |  | 1.500 |
|  | 5% | 100% |  |  | 1.500 |
|  | 100% | 99.99% |  |  | 1.492 |
| 50% | 100% | 99.90% | 4,654 | 1.500 | 1.430 |
|  | 100% | 99.80% |  |  | 1.378 |
|  | 88.71% | 99.80% |  |  | 1.366 |
|  | 82.26% | 100% |  |  | 1.500 |
|  | 5% | 100% |  |  | 1.500 |
|  | 100% | 99.99% |  |  | 1.493 |
| 25% | 100% | 99.90% | 2,586 | 1.500 | 1.437 |
|  | 100% | 99.80% |  |  | 1.388 |
|  | 88.71% | 99.80% |  |  | 1.377 |
|  | 82.26% | 100% |  |  | 1.500 |
|  | 5% | 100% |  |  | 1.500 |
|  | 100% | 99.99% |  |  | 1.493 |
| 10% | 100% | 99.90% | 1,108 | 1.500 | 1.440 |
|  | 100% | 99.80% |  |  | 1.393 |
|  | 88.71% | 99.80% |  |  | 1.382 |
|  | 82.26% | 100% |  |  | 1.500 |
| The table envisions a cohort study that observes the relationship between factors that have a cancer prevalence and a risk ratio of 1.5. It shows the change in the risk ratio for each assumption of matching accuracy. For 100,000 observer-years, a four-step factor prevalence was set and the number of observation events was determined so that the risk ratio was approximately 1.5. The prevalence of cancer was 775.7 / 100,000 person-years based on the latest prevalence in Japan. The gray background shows estimates using data from the experiment. | | | | | |

| Table A4. The effect of matching accuracy on the estimation of the odds ratio given by a factor in a case-control study | | | | | | | | | | |
| --- | --- | --- | --- | --- | --- | --- | --- | --- | --- | --- |
| Assumptions of  matching accuracy | |  | Exposure rate | | | | |  | Odds ratio | |
| Sensitivity | Specificity |  | Population | Case | | Control | |  | True | Estimate |
|  |  |  |  | True | Estimate | True | Estimate |  |  |  |
| 90% | 100% |  |  |  | NA |  | 73.28% |  |  | 2.967 |
| 85% | 100% |  |  |  | NA |  | 73.39% |  |  | 2.951 |
| 100% | 99.90% |  | 75% | 89.06% | 88.94% | 73.07% | NA |  | 3.000 | 2.965 |
| 100% | 99.80% |  |  |  | 88.83% |  | NA |  |  | 2.931 |
| 88.71% | 99.80% |  |  |  | 88.80% |  | 73.31% |  |  | 2.886 |
| 82.26% | 100% |  |  |  | 89.06% |  | 73.45% |  |  | 2.942 |
| 90% | 100% |  |  |  | NA |  | 47.24% |  |  | 2.958 |
| 85% | 100% |  |  |  | NA |  | 47.41% |  |  | 2.938 |
| 100% | 99.90% |  | 50% | 72.59% | 72.41% | 46.89% | NA |  | 3.000 | 2.972 |
| 100% | 99.80% |  |  |  | 72.22% |  | NA |  |  | 2.945 |
| 88.71% | 99.80% |  |  |  | 72.18% |  | 47.28% |  |  | 2.892 |
| 82.26% | 100% |  |  |  | 72.59% |  | 47.50% |  |  | 2.927 |
| 90% | 100% |  |  |  | NA |  | 22.43% |  |  | 2.944 |
| 85% | 100% |  |  |  | NA |  | 22.59% |  |  | 2.918 |
| 100% | 99.90% |  | 25% | 45.99% | 45.82% | 22.11% | NA |  | 3.000 | 2.979 |
| 100% | 99.80% |  |  |  | 45.65% |  | NA |  |  | 2.959 |
| 88.71% | 99.80% |  |  |  | 45.61% |  | 22.48% |  |  | 2.892 |
| 82.26% | 100% |  |  |  | 45.99% |  | 22.68% |  |  | 2.903 |
| 90% | 100% |  |  |  | NA |  | 8.58% |  |  | 2.932 |
| 85% | 100% |  |  |  | NA |  | 8.67% |  |  | 2.899 |
| 100% | 99.90% |  | 10% | 21.59% | 21.49% | 8.41% | NA |  | 3.000 | 2.983 |
| 100% | 99.80% |  |  |  | 21.40% |  | NA |  |  | 2.967 |
| 88.71% | 99.80% |  |  |  | 21.37% |  | 8.61% |  |  | 2.887 |
| 82.26% | 100% |  |  |  | 21.59% |  | 8.72% |  |  | 2.882 |
| The table assumes a case-control study investigating the relationship between the onset of diabetes and factors with an odds ratio of 3. It shows the change in the odds ratio for each assumption of matching accuracy. It was set to collect cases and non-cases at a ratio of 1: 2. The number of cases was 1,000, the size of the population was 10,000,000, and the prevalence was set at 12.1%, based on data on the prevalence of the disease among Japanese people. The gray background shows estimates using data from the experiment. NA = not affected. | | | | | | | | | | |

| Table A5. The effect of matching accuracy on the estimation of the odds ratio given by a factor in a case-control study | | | | | | | | | | |
| --- | --- | --- | --- | --- | --- | --- | --- | --- | --- | --- |
| Assumptions of  matching accuracy | |  | Exposure rate | | | | |  | Odds ratio | |
| Sensitivity | Specificity |  | Population | Case | | Control | |  | True | Estimate |
|  |  |  |  | True | Estimate | True | Estimate |  |  |  |
| 90% | 100% |  |  |  | NA |  | 74.99% |  |  | 2.999 |
| 85% | 100% |  |  |  | NA |  | 74.99% |  |  | 2.999 |
| 100% | 99.90% |  | 75% | 89.99% | 82.49% | 74.98% | NA |  | 3.000 | 1.572 |
| 100% | 99.80% |  |  |  | 79.99% |  | NA |  |  | 1.334 |
| 88.71% | 99.80% |  |  |  | 79.60% |  | 74.99% |  |  | 1.301 |
| 82.26% | 100% |  |  |  | 89.99% |  | 74.99% |  |  | 2.999 |
| 90% | 100% |  |  |  | NA |  | 49.98% |  |  | 3.000 |
| 85% | 100% |  |  |  | NA |  | 49.98% |  |  | 2.999 |
| 100% | 99.90% |  | 50% | 74.98% | 62.48% | 49.97% | NA |  | 3.000 | 1.667 |
| 100% | 99.80% |  |  |  | 58.32% |  | NA |  |  | 1.400 |
| 88.71% | 99.80% |  |  |  | 57.66% |  | 49.98% |  |  | 1.363 |
| 82.26% | 100% |  |  |  | 74.98% |  | 49.98% |  |  | 2.999 |
| 90% | 100% |  |  |  | NA |  | 24.98% |  |  | 3.000 |
| 85% | 100% |  |  |  | NA |  | 24.98% |  |  | 3.000 |
| 100% | 99.90% |  | 25% | 49.97% | 37.48% | 24.98% | NA |  | 3.000 | 1.801 |
| 100% | 99.80% |  |  |  | 33.31% |  | NA |  |  | 1.501 |
| 88.71% | 99.80% |  |  |  | 32.66% |  | 24.98% |  |  | 1.457 |
| 82.26% | 100% |  |  |  | 49.97% |  | 24.98% |  |  | 3.000 |
| 90% | 100% |  |  |  | NA |  | 9.99% |  |  | 3.000 |
| 85% | 100% |  |  |  | NA |  | 9.99% |  |  | 2.999 |
| 100% | 99.90% |  | 10% | 24.97% | 17.48% | 9.99% | NA |  | 3.000 | 1.910 |
| 100% | 99.80% |  |  |  | 14.98% |  | NA |  |  | 1.589 |
| 88.71% | 99.80% |  |  |  | 14.59% |  | 9.99% |  |  | 1.540 |
| 82.26% | 100% |  |  |  | 24.97% |  | 9.99% |  |  | 2.999 |
| The table assumes a case-control study investigating the relationship between the onset of ulcerative colitis and factors with an odds ratio of 3. It shows the change in the odds ratio for each assumption of matching accuracy. It was set to collect cases and non-cases at a ratio of 1: 2. The number of cases was 1000, the size of the population was 10 million, and the prevalence was 0.1% based on the prevalence in Japanese. The gray background shows estimates using data from the experiment. NA = not affected. | | | | | | | | | | |
